# Report from the 3rd Cardiovascular Outcome Trial (CVOT) Summit of the Diabetes & Cardiovascular Disease (D&CVD) EASD Study Group

**DOI:** 10.1186/s12933-018-0667-2

**Published:** 2018-02-19

**Authors:** Oliver Schnell, Eberhard Standl, Doina Catrinoiu, Stefano Genovese, Nebojsa Lalic, Katarina Lalic, Jan Skrha, Paul Valensi, Antonio Ceriello

**Affiliations:** 1 Forschergruppe Diabetes e.V., Munich, Ingolstaedter Landstrasse 1, Neuherberg, 85764 Munich, Germany; 2 Internal Medicine Department, Clinical County Emergency Hospital Constanta, Tomis Blvd. No. 145, 900591 Constanta, Romania; 3 Diabetes, Endocrine and Metabolic Disease Unit, IRCCS Centro Cardiologico Monzino, Via Carlo Parea 4, 20138 Milan, Italy; 4 Clinic for Endocrinology, Diabetes and Metabolic Diseases, Clinical Center of Serbia, Faculty of Medicine, University of Belgrade, Dr Subotica 13, Belgrade, 11000 Serbia; 5 3rd Department of Internal Medicine, Charles University, 1st Faculty of Medicine, U Nemocnice 1, Prague 2, 128 08 Czech Republic; 6 Department of Endocrinology Diabetology Nutrition, Paris 13 University, CINFO, CRNH-IdF, Jean VERDIER Hospital, Avenue du 14 Juillet, 93140 Bondy, France; 7 Institut d’Investigacions Biomèdiques August Pi i Sunyer (IDIBAPS) and Centro de Investigación Biomedica en Red de Diabetes y Enfermedades Metabólicas Asociadas (CIBERDEM), Barcelona, Spain; 8 Department of Cardiovascular and Metabolic Diseases, IRCCS MultiMedica, Via Milanese 300, 20099 Sesto San Giovanni, MI Italy

**Keywords:** CVOT Summit, D&CVD EASD Study Group, Diabetes, Cardiovascular risk, CVOT, DEVOTE, CANVAS program, EXSCEL, ACE

## Abstract

The 3rd Cardiovascular Outcome Trial Summit of the Diabetes & Cardiovascular Disease EASD Study Group was held on the 26–27 October 2017 in Munich. As in 2015 and 2016, this summit was organised in light of recently completed and published CVOTs on diabetes, aiming to serve as a reference meeting for in-depth discussions on the topic. Amongst others, the CVOTs EXSCEL, DEVOTE, the CANVAS program and the ACE-trial, which released primary outcome results in 2017, were discussed. Trial implications for diabetes management and recent perspectives of diabetologists, cardiologists, endocrinologists, nephrologists and general practitioners were highlighted. The clinical relevance of cardiovascular outcome trials and its implications regarding reimbursement were compared with real-world studies. The 4th Cardiovascular Outcome Trial Summit will be held in Munich 25–26 October 2018 (http://www.dcvd.org).

## Background

Patients with diabetes have an up to 50% increased risk of cardiovascular (CV) disease (CVD) compared to individuals without diabetes [1]. Optimisation of glycaemic control can reduce the long-term development of CVD and mortality [2, 3].

Driven by the Food and Drug Administration (FDA) and the European Medicines Agency (EMA), guidelines for the approval of novel glucose-lowering medications were published in 2008 and 2012, respectively [4, 5]. These guidelines highlight, that new therapeutic approaches should not result in an increased CV risk. To rule out CV harm of novel treatment approaches, CV safety and benefits of glucose-lowering medications have therefore been the focus of cardiovascular outcome trials (CVOTs) in diabetes, which have been performed according to the suggestions of the abovementioned guidelines. In the CVOTs, combined primary CV endpoints are evaluated, which include CV mortality, non-fatal myocardial infarction (MI) and non-fatal stroke (3-point-MACE). Additional components could be e.g. hospitalisation for heart failure (HF), acute coronary syndrome and revascularisation procedures. Several CVOTs, which analysed DPP-4 inhibitors (saxagliptin, alogliptin, sitagliptin), GLP-1 receptor agonists (RA; lixisenatide, liraglutide, semaglutide) and SGLT-2 inhibitors (empagliflozin), were published until 2016 [6–12].

In 2017, CVOTs for canagliflozin (SGLT-2 inhibitor, CANVAS program), exenatide once weekly (GLP-1 RA, EXSCEL) and Insulin degludec (basal insulin analogue, DEVOTE) were published [13–16]. Also the ACE trial (acarbose), which focused on secondary prevention of CVD, was completed [17]. Other CVOTs, which included significant numbers of diabetic patients, investigated lipid-lowering strategies with evolocumab (FOURIER) and anacetrapib (REVEAL) [18, 19]. As in 2015 and 2016 [20, 21], we present and summarise key aspects, which were discussed at the 3^rd^ CVOT Summit.

### Updates on CVOTs

A summary of characteristics and results of CVOTs from 2017 is presented in Tables 1 and 2.

**Table 1:** Overview of basic characteristics of CVOTs completed 2016/17 and published in 2017

	Study status	Drug	Drug class	Intervention	Primary outcome	N	Follow-up (years)	Start and end date	Clinicaltrials.gov ID
CANVAS program	Completed	Canagliflozin	SGLT-2 Inhibitor	Canagliflozin 100 mg vs. canagliflozin 300 mg vs. placebo	CV death, MI, or stroke	10,142	3.6	12.2009–02.2017	NCT01032629
EXSCEL	Completed	Exenatide	GLP-1 receptor agonist	Exenatide once-weekly vs. placebo	CV death, MI, or stroke	14,752	3.2	06.2010–04.2017	NCT01144338
DEVOTE	Completed	Insulin degludec	Basal insulins	Insulin degludec vs. Insulin glargine	CV death, MI, or stroke	7637	2.0	10.2013–10.2016	NCT01959529
ACE	Completed	Acarbose	α-glucosidase inhibitor	Acarbose vs. placebo	CV death, MI, or stroke, HHF,HUA	6522	5.0	02.2009–04.2017	NCT00829660
FOURIER	Completed	Evolocumab	PCSK9 inhibitor	Evolocumab vs. placebo	CV death, MI, stroke, UA or coronary revascularisation	27,564	2.2	01.2013–11.2016	NCT01764633

**Table 2:** CVOTs completed in 2016/17 and published in 2017: comparison of results vs. placebo

	CANVAS program [13]		EXSCEL [14]		DEVOTE [15]		ACE [17]		FOURIER [18]	
Cardiovascular endpoints	Class	HR (95% CI) p value	Class	HR (95% CI) p value	Class	HR (95% CI) p value	Class	HR (95% CI) p value	Class	HR (95% CI) p value
Primary Composite MACE	CV death, MI or stroke	0.86 (0.75–0.97) 0.02^a^	CV death, MI or stroke	0.91 (0.83–1.00) 0.06^a^	CV death, MI or stroke	0.91 (0.78–1.06) <0.001	CV death, MI, stroke, UA and HF	0.98 (0.86–1.11) 0.73	CV death, MI, stroke, UA and coronary re-vascularisation	0.85 (0.79–0.92) <0.001
Cardiovascular death	Primary endpoint	0.87 (0.72–1.06) –	Secondary endpoint	0.88 (0.76–1.02) –	Primary endpoint	0.96 (0.76–1.21) 0.71	Secondary endpoint	0.89 (0.71–1.11) 0.29	Secondary endpoint	1.05 (0.88–1.25) 0.62
Myocardial infarction	Primary endpoint	0.89 (0.73–1.09) –	Secondary endpoint	0.97 (0.85–1.10) –	Primary endpoint	0.85 (0.68–1.06) 0.15	Secondary endpoint	1.12 (0.87–1.46) 0.38	Secondary endpoint	0.73 (0.65–0.82) <0.001
Stroke	Primary endpoint	0.87 (0.69–1.09) –	Secondary endpoint	0.85 (0.70–1.03) –	Primary endpoint	0.90 (0.65–1.23) 0.50	Secondary endpoint	0.97 (0.70–1.33) 0.83	Secondary endpoint	0.79 (0.66–0.95) 0.01
Hospitalisation for unstable angina	–	– –	–	– –	Primary endpoint	0.95 (0.68–1.31) 0.74	Secondary endpoint	1.02 (0.82–1.26) 0.87	Secondary endpoint	0.99 (0.82–1.18) 0.89
Hospitalisation for heart failure	Secondary endpoint	0.67 (0.52–0.87) –	Secondary endpoint	0.94 (0.78–1.13) –	–	– –	Secondary endpoint	0.89 (0.63–1.24) 0.48	–	– –

	Event rate (%) active group		Event rate (%) active group		Event rate (%) active group		Event rate (%) active group		Event rate (%) active group	
Primary composite MACE	26.9^b^		11.4		8.5		14.4		9.8	

	No. (%) p-value		No. (%) p-value		No. (%) p-value		No. (%) p-value		No. (%) p-value	
Renal event	19.7^b^ 0.32		55 (0.7) –		3.8 (–) –		– (–) –		– (–) –	
Acute pancreatitis	0.5^b^ 0.63		26 (0.4) –		– (–) –		– (–) –		– (–) –	
Hypoglycaemia events	50.0^b^ 0.20		247 (3.4)^c^ –		4.9 (–)^c^ <0.001^a^		54 (2)^c^ 0.95		– (–) –	

^a^Superiority test

^b^Number of participants per 1000 patient-year

^c^Severe hypoglycaemia events

### SGLT-2 inhibitors

The CANVAS program was a combination of two trials involving 10,142 participants with type 2 diabetes mellitus (T2DM) at high CV risk. It was shown that patients treated with the SGLT-2 inhibitor canagliflozin had a lower risk of CV events and a significant reduction of hospitalisation for HF. Although on the basis of the pre-specified hypothesis testing sequence the renal outcomes are not viewed as statistically significant, the results showed a possible benefit of canagliflozin with respect to the progression of albuminuria and the composite outcome of a sustained 40% reduction in the estimated glomerular filtration rate (eGFR), the need for renal-replacement therapy or death from renal cause. The risk for amputation was increased compared to the control group [13].

### GLP-1 receptor agonists

The results from the EXSCEL trial demonstrated CV safety in high risk subjects with T2DM who were treated with long-acting exenatide once weekly. With respect to safety the GLP-1 RA was non-inferior to placebo but not superior with respect to efficacy. The secondary outcomes were consistent with the primary outcome: CVD, fatal or nonfatal MI, fatal or nonfatal stroke as well as hospitalisation for HF and for acute coronary syndrome did not differ significantly between both groups [14]. There was, however, a 14% reduction in all-cause mortality with exenatide once weekly versus placebo (hazard ratio 0.86, 95% CI 0.77–0.97), although this difference could not be rated as significant due to the protocol-defined hierarchical order of statistical testing.

### Basal insulin

Results of the DEVOTE trial indicate that for T2DM treatment, the ultra-long-acting, once-daily basal Insulin degludec is as safe in CV terms as Insulin glargine and associated with much lower rates of severe hypoglycaemia. Treatment with Insulin degludec resulted in a 27% reduction in the number of patients experiencing a severe episode, resulting in a 40% overall reduction of total episodes. Patients treated with Insulin degludec also experienced a 53% reduction in the number of nocturnal episodes of severe hypoglycaemia [15].

### Alpha-glucosidase inhibitors

In the ACE trial, pre-existing diabetes was an exclusion criterion and for inclusion participants required a history of CVD and impaired glucose tolerance. Designed as a CVOT with a 3-point-MACE, the ACE study could not achieve enough events, yielding a shift to an expanded 5-point-MACE [adding hospitalisation for HF and unstable angina (UA)] as primary outcome. The rate of events of the primary endpoint was not reduced, comparing acarbose with placebo. Also, most secondary outcomes did not show a difference between the two treatment groups. Confirming the primary result of the Stop-NIDDM trial [22], however, the number of participants who developed diabetes was reduced by 18% in the acarbose group compared to the placebo group [17].

### Lipid-lowering strategies

The FOURIER trial demonstrated, that the PCSK9 inhibitor evolocumab on top of optimised statin therapy can reduce LDL-cholesterol to an unprecedented low level that was associated with a reduction of CV events. The positive outcomes on CV risk were especially apparent in patients with diabetes, though no effects on glucose level or HbA1c were observed. In contrast to statins, evolocumab did not increase the risk of new onset of diabetes, but this effect might be concealed by the relatively short follow-up time [18].

Even though the REVEAL trial provided positive results on another lipid-modifying medication—the CETP inhibitor anacetrapib—that reduced LDL-cholesterol and increased HDL-cholesterol levels, with a 9% significant reduction in 3-point-MACE, the small increase in blood pressure and minor reduction in kidney function prevented the filing for approval by the FDA [19].

### Real-world data

Apart from CVOTs the establishment of “real-world” data is an emerging field in diabetes. Regulatory bodies like FDA, the National Institute for Health and Care Excellence (NICE) or the German “Institut für Qualität und Wirtschaftlichkeit im Gesundheitswesen” (IQWiG) are demanding an increased inclusion and implementation of real world data to complement results of CVOTs. Studies based on electronic health records and registries could advance translational research and are often seen as a cost saving opportunity to improve patient care and population health.

Two examples concerning database-analysis were presented at the 3^rd^ CVOT Summit: CVD-REAL assessed data from more than 300,000 T2DM patients across six countries, 87% of whom did not have a history of CVD. These observational data showed that across this broad population treatment with SGLT-2 inhibitors (canagliflozin, dapagliflozin and empagliflozin) was significantly associated with a reduced overall rate of hospitalisation for HF by 39% and death from any cause by 51%, compared to other treatment approaches. For the composite endpoint of hospitalisation for HF and death from any cause, a reduction of 46% was observed [23]. However, bias by drug indication cannot be totally excluded in CVD-REAL, as no clinical chemistry data were available for most patients and results could not be stratified for eGFR [23].

Data from the Swedish RiksSvikt Heart Failure registry from 2003 to 2011 was also presented. It included more than 36,000 patients with HF, of whom 8809 presented with diabetes. The database recorded about 70 variables and treatments managed by the Uppsala Clinical Research Centre. Data analysis revealed amongst others that diabetes compromises survival in HF irrespective of sex, HF aetiology or type. It increases mortality by 30–70% in diabetic patients. The prognosis is comparable between male and female diabetes patients and worst in those with systolic dysfunction and ischemic heart disease. It was concluded from the registry that special attention is required for diabetic patients with HF and strategies for improvement of outcome need to be established [24–26].

It was agreed that real-world evidences are beneficial to increase the understanding of the course of diabetes and its complications. A prerequisite for utilisation and interpretation of real-world data are high data quality and integrity of cases within the studied population. Nevertheless, discrepancies between randomised control trials and real-world data impede the comparison of such results. It was discussed that adherence to medication might differ between the two study designs and thereby contribute to contrasting results [27]. It was consensus that real-world data are valuable tools to complement, but not replace CVOTs. Nevertheless the role for real-world data will increase in the future.

### Key questions discussed during the 3rd CVOT Summit: How should results of CVOTs be reflected in guidelines?

The requirement for a balanced reflection of CVOT results in guidelines is undoubted. It was agreed that both national and international guidelines need to incorporate CVOT results in a swift and comprehensive manner, and that an adaption of international guidelines supports updates of guidelines on a local level [28]. Further strategies to distribute new CVOT results may also include online publications of guidelines and regular updates of medical standards.

In the future guidelines might need to distinguish between diabetic patients with and without CVD considering separate algorithms for treatment strategies of the two populations. Updated guidelines should not only reflect on medication that has demonstrated CV safety but also include examples of CV risk reduction. Updated guidelines should clearly discuss potential class effects of GLP-1 RAs, DPP-4 and SGLT-2 inhibitors, while at the same time highlight drug-specific effects observed in the outcome trials. Side effects and adverse events need to be included.

It was frequently suggested to provide joint guidelines by and for diabetologists, cardiologists and general practitioners (GPs).

### CVOTs in diabetes: are we looking at the right endpoints?

Considering CVOTs from both a medical and regulatory perspective, the primary endpoint (MACE) was considered appropriate to demonstrate CV safety. This primary endpoint can be supported by more detailed secondary endpoints like time to first hospitalisation for HF or first occurrence of a microvascular event. Depending on the CVOT approach, additional, more specific endpoints may be considered. It has to be acknowledged that the robustness of some endpoints, e.g. the occurrence of an acute coronary syndrome, is still debated. Other endpoints like the occurrence and progression of diabetic retinopathy still need to be standardised to enable comparison between study outcomes. Overall, it was consensus that a clearer delineation of safety and efficacy components as well as better control of type 1 errors are required and that the implementation and publication of CVOTs should be standardised.

### Will diabetes with CVD in the future be treated by general practitioners, diabetologists, cardiologists or nephrologists?

The general consensus was that comprehensive diabetes management is best to be performed by both diabetologists and GPs. Cooperation with cardiologists and nephrologists still needs to be optimised to deliver evidence-based medicine as a multi-disciplinary approach. Additionally, neurologists, psychiatrists, ophthalmologists and other specialities should be associated with the treatment of diabetes more closely. Thus, cross-functional medical education should be enhanced and guideline across associations should be aligned. Treatment decisions and recommendations should be shared more intensively between medical specialties and the primary care sector. In general, this was considered an important opportunity for establishing a network of cross-specialist collaborations in health care systems.

### Should we continue CV trials in the light of approved benefits?

In general, the answer to this question was affirmative. Any medication for chronic disease should undergo long-term safety studies best carried out with standardised and clinically relevant endpoints. An additional CV or microvascular benefit was considered to be a pre-requisite for new approaches in the management of diabetes. Beside the high relevance of CV endpoints, microvascular endpoints should not be neglected. Studies focussing primarily on renal outcomes in diabetes are awaited in the near future.

Suggestions for future CVOTs were discussed. It was agreed that novel treatments should also be tested against treatment approaches, which have already shown to have proven CV benefit. It was acknowledged that it will be an increasing challenge to demonstrate an additional CV benefit. Suggestions included the need for mechanistic studies to further understand mechanisms responsible for CV and/or microvascular benefits as well as potential side effects. Studies should also help to clarify which group of diabetic patients benefits from specific treatment strategies or class of treatment and which diabetic patient groups do not.

## Conclusion

The 3rd CVOT Summit of the D&CVD EASD Study Group discussed key results of recent CVOTs and new studies on lipid-lowering strategies in an interactive multi-disciplinary format. The summit discussed both potentials and limitations of current CVOT designs as well as strategies for the implementation of CVOT results in treatment guidelines. It also discussed potential elements for future design of CVOTs in diabetes and shaped the role for real-world data. The D&CVD EASD Study Group will continue its activity. In-depth discussions and presentations of upcoming CVOTs like CAROLINA, REWIND and DECLARE-TIMI, will be resumed at the 4th CVOT Summit, which will be held from 25 – 26 October 2018 in Munich (http://www.dcvd.org).

## Abbreviations

CV: cardiovascular
CVD: cardiovascular disease
CVOT: cardiovascular outcome trials
D&CVD: Diabetes and Cardiovascular Disease
EASD: European Association of the Study of Diabetes
eGFR: estimated glomerular filtration rate
EMA: European Medicines Agency
FDA: Food and Drug Administration
GP: general practitioner
HF: heart failure
HHF: hospitalisation for heart failure
HUA: hospitalisation for unstable angina
IQWiG: Institut für Qualität und Wirtschaftlichkeit im Gesundheitswesen
MI: myocardial infarction
NICE: National Institute for Health and Care Excellence
RA: receptor agonist
T2DM: type 2 diabetes mellitus
UA: unstable angina
